# Supplementary material for: Comparative analyses of eighteen rapid antigen tests and RT-PCR for COVID-19 quarantine and surveillance-based isolation
Source: Commun Med (Lond). 2022 Jul 9;2:84. doi: 10.1038/s43856-022-00147-y (PMC9271059; doi:10.1038/s43856-022-00147-y)
Supplement: Supplementary file 1 — Description of Additional Supplementary Files [file 43856_2022_147_MOESM1_ESM.pdf]

Description of Additional Supplementary Files

File Name: Supplementary Data 1

Description: The percent positive agreement data for the rapid antigen tests based on the day of symptom onset.

File Name: Supplementary Data 2

Description: Estimated coefficients and 95% credible intervals (generated from 1,000 samples) for the logistic regression models describing the percent positive agreement curve for each of the rapid antigen tests.

File Name: Supplementary Data 3

Description: The specificity of RT-PCR and the EUA rapid antigen tests and 95% credible intervals constructed from 1000 samples.

File Name: Supplementary Data 4

Description: Specifying a 4.4-day incubation period, a log-Normal distribution for the temporal RT-PCR diagnostic sensitivity, a basic reproductive number of 3.2, and 35.1% of infections being asymptomatic, the required quarantine durations, serial testing frequencies, and probabilities of false-positives for each serial testing frequency with 95% credible intervals.

File Name: Supplementary Data 5

Description: Specifying a 5.72-day incubation period, a log-Normal distribution for the temporal RT-PCR diagnostic sensitivity, a basic reproductive number of 3.2, and 35.1% of infections being asymptomatic, the required quarantine durations, serial testing frequencies, and probabilities of false-positives associated with the serial testing frequency with 95% credible intervals.

File Name: Supplementary Data 6

Description: The change in the required quarantine durations and serial testing frequencies when there is a specified threshold in which the rapid antigen test can return a positive test result relative to the results in Supplementary Data 4 for the maximum likelihood estimates.

File Name: Supplementary Data 7

Description: Specifying a 4.4-day incubation period, a log-Student's t distribution for the temporal RT-PCR diagnostic sensitivity, a basic reproductive number of 3.2, and 35.1% of infections being asymptomatic, the required quarantine durations, serial testing frequencies, and probabilities of false-positives for each serial testing frequency with 95% credible intervals.
