# Supplementary material for: Comparative analyses of eighteen rapid antigen tests and RT-PCR for COVID-19 quarantine and surveillance-based isolation
Source: Commun Med (Lond). 2022 Jul 9;2:84. doi: 10.1038/s43856-022-00147-y (PMC9271059; doi:10.1038/s43856-022-00147-y)
Supplement: Supplementary file 3 — Supplementary Data 1 [file 43856_2022_147_MOESM3_ESM.pdf]

<table>
<tr><th rowspan="2">Test name</th><th colspan="16">Days since symptom onset</th></tr>
<tr><th>0</th><th>1</th><th>2</th><th>3</th><th>4</th><th>5</th><th>6</th><th>7</th><th>8</th><th>9</th><th>10</th><th>11</th><th>12</th><th>13</th><th>14</th><th>>14</th></tr>
<tr><td>BD Veritor [a,b,c]</td><td>•</td><td>7/8</td><td>10/12</td><td>1/2</td><td>5/5</td><td>3/4</td><td>2/3</td><td>1/4</td><td>•</td><td>•</td><td>•</td><td>•</td><td>•</td><td>•</td><td>•</td><td>•</td></tr>
<tr><td>BinaxNOW [b,c]</td><td>•</td><td>10/12</td><td>18/22</td><td>13/16</td><td>9/13</td><td>13/15</td><td>12/12</td><td>24/27</td><td colspan="3">19/27</td><td colspan="4">8/17</td><td>3/6</td></tr>
<tr><td>BinaxNOW [c,d]</td><td>7/8</td><td>30/33</td><td>30/32</td><td>22/22</td><td>8/9</td><td>6/6</td><td>7/7</td><td>2/2</td><td colspan="8">12/17</td></tr>
<tr><td>CareStart [b,c]</td><td>3/3</td><td>7/8</td><td>11/13</td><td>8/9</td><td>3/4</td><td>2/2</td><td>•</td><td>•</td><td>•</td><td>•</td><td>•</td><td>•</td><td>•</td><td>•</td><td>•</td><td>•</td></tr>
<tr><td>CareStart [b,e]</td><td>•</td><td>•</td><td>7/7</td><td>8/8</td><td>7/8</td><td>8/9</td><td>•</td><td>•</td><td>•</td><td>•</td><td>•</td><td>•</td><td>•</td><td>•</td><td>•</td><td>•</td></tr>
<tr><td>CareStart [c,d]</td><td>4/5</td><td>7/9</td><td>8/9</td><td>11/12</td><td>6/8</td><td>3/3</td><td>3/4</td><td>1/1</td><td colspan="8">2/9</td></tr>
<tr><td>Celltrion DiaTrust [b,e]</td><td>•</td><td>3/3</td><td>6/6</td><td>5/6</td><td>9/9</td><td>2/2</td><td>1/1</td><td>2/3</td><td>•</td><td>•</td><td>•</td><td>•</td><td>•</td><td>•</td><td>•</td><td>•</td></tr>
<tr><td>Clip COVID [b,c]</td><td>•</td><td>4/5</td><td>12/12</td><td>8/8</td><td>6/6</td><td>1/1</td><td>•</td><td>•</td><td>•</td><td>•</td><td>•</td><td>•</td><td>•</td><td>•</td><td>•</td><td>•</td></tr>
<tr><td>Ellume [b,f]</td><td colspan="2">7/7</td><td>6/6</td><td>5/5</td><td>3/3</td><td>1/1</td><td>3/3</td><td>0/1</td><td>•</td><td>•</td><td>•</td><td>•</td><td>•</td><td>•</td><td>•</td><td>•</td></tr>
<tr><td>Liaison [b,c]</td><td>6/6</td><td>5/6</td><td>2/2</td><td>2/2</td><td>0/0</td><td>2/2</td><td>4/4</td><td>1/1</td><td>4/4</td><td>4/4</td><td>•</td><td>•</td><td>•</td><td>•</td><td>•</td><td>•</td></tr>
<tr><td>Liaison [b,e]</td><td>8/9</td><td>9/9</td><td>4/4</td><td>10/10</td><td>5/6</td><td>3/3</td><td>3/3</td><td>3/3</td><td>1/1</td><td>2/2</td><td>1/1</td><td>•</td><td>•</td><td>•</td><td>•</td><td>•</td></tr>
<tr><td>LumiraDx [b,c]</td><td>6/6</td><td>6/6</td><td>16/16</td><td>9/9</td><td>17/18</td><td>6/6</td><td>6/6</td><td>6/6</td><td>2/2</td><td>0/0</td><td>2/2</td><td>3/3</td><td>2/3</td><td>•</td><td>•</td><td>•</td></tr>
<tr><td>LumiraDx [b,e]</td><td>2/2</td><td>4/4</td><td>3/3</td><td>8/8</td><td>5/5</td><td>1/1</td><td>3/3</td><td>8/8</td><td>2/2</td><td>0/0</td><td>2/3</td><td>1/1</td><td>•</td><td>•</td><td>•</td><td>•</td></tr>
<tr><td>Omnia [b,c]</td><td>1/1</td><td>5/5</td><td>7/9</td><td>11/13</td><td>19/21</td><td>2/2</td><td>6/6</td><td>•</td><td>•</td><td>•</td><td>•</td><td>•</td><td>•</td><td>•</td><td>•</td><td>•</td></tr>
<tr><td>SCoV-2 Ag Detect [b,c]</td><td>•</td><td>7/8</td><td>5/6</td><td>14/16</td><td>10/11</td><td>3/4</td><td>•</td><td>•</td><td>•</td><td>•</td><td>•</td><td>•</td><td>•</td><td>•</td><td>•</td><td>•</td></tr>
<tr><td>Simoa [b,e]</td><td>8/8</td><td>14/15</td><td>11/11</td><td>10/10</td><td>9/9</td><td>4/4</td><td>4/4</td><td>8/8</td><td colspan="7">18/19</td><td>•</td></tr>
<tr><td>Sofia [b,c]</td><td>•</td><td>5/5</td><td>11/12</td><td>3/3</td><td>5/5</td><td>2/2</td><td>2/2</td><td>1/1</td><td>•</td><td>•</td><td>•</td><td>•</td><td>•</td><td>•</td><td>•</td><td>•</td></tr>
<tr><td rowspan="2">Sofia [c,d]</td><td>1/1</td><td>6/6</td><td>5/6</td><td>7/7</td><td>1/2</td><td>•</td><td>•</td><td>•</td><td>3/3</td><td>•</td><td>•</td><td>•</td><td>•</td><td>•</td><td>•</td><td>•</td></tr>
<tr><td colspan="6">3/9</td><td colspan="10">5/5</td></tr>
<tr><td>Sofia 2 Flu + SARS [b,e]</td><td>0/0</td><td>14/14</td><td>10/11</td><td>4/4</td><td>10/10</td><td>2/3</td><td>•</td><td>•</td><td>•</td><td>•</td><td>•</td><td>•</td><td>•</td><td>•</td><td>•</td><td>•</td></tr>
<tr><td>Status COVID-19/Flu [b,e]</td><td>16/18</td><td>13/13</td><td>6/6</td><td>6/6</td><td>5/5</td><td>0/1</td><td>•</td><td>•</td><td>•</td><td>•</td><td>•</td><td>•</td><td>•</td><td>•</td><td>•</td><td>•</td></tr>
<tr><td>Vitros [b,e]</td><td>4/8</td><td>2/2</td><td>4/4</td><td>6/6</td><td>0/0</td><td>2/2</td><td>3/4</td><td>3/4</td><td>•</td><td>•</td><td>•</td><td>•</td><td>•</td><td>•</td><td>•</td><td>•</td></tr>
</table>

• Indicates that there was no data available for the specified time period
[a] Peer-reviewed
[b] Data from EUA submission
[c] Anterior nasal swab
[d] Data from community testing
[e] Nasopharyngeal swab
[f] Mid-turbinate swab
