# Supplementary material for: Comparative analyses of eighteen rapid antigen tests and RT-PCR for COVID-19 quarantine and surveillance-based isolation
Source: Commun Med (Lond). 2022 Jul 9;2:84. doi: 10.1038/s43856-022-00147-y (PMC9271059; doi:10.1038/s43856-022-00147-y)
Supplement: Supplementary file 4 — Supplementary Data 2 [file 43856_2022_147_MOESM4_ESM.pdf]

Test name	β_0	β_1
BD Veritor [a,b,c]	2.55 (1.1–4.68)	− 0.379 (− 0.852–− 0.0609)
BinaxNOW [b,c]	1.95 (1.25–2.85)	− 0.1 (− 0.214–− 0.0066)
BinaxNOW [c,d]	2.91 (2.2–3.81)	-1.93×10^{-9} (-1.03×10^{-8}–0)
BinaxNOW [c,e]	2.5 (1.92–3.17)	− 0.152 (− 0.247–− 0.0612)
CareStart [b,c]	2.2 (1.39–4.02)	− 0.123 (− 0.616–− 0.00178)
CareStart [b,f]	6.77 (2.16–20)	− 0.998 (− 3.7–0)
CareStart [c,d]	1.87 (1.18–2.69)	-2.19×10^{-11} (-1.16×10^{-10}–0)
CareStart [c,e]	1.89 (1.35–2.51)	-4.9×10^{-10} (-2.67×10^{-9}–-2.04×10^{-11})
Celltrion DiaTrust [b,f]	4.81 (2.19–11.5)	− 0.518 (− 1.66–− 0.0217)
Clip COVID [b,c]	3.43 (1.77–6.74)	-1.28×10^{-8} (-8.08×10^{-8}–-2.59×10^{-9})
Ellume [b,g]	100 (100–490)	− 15.3 (− 59.3–− 3.6)
Liaison [b,c]	3.47 (1.85–6.82)	-1.15×10^{-9} (-6.05×10^{-9}–0)
Liaison [b,f]	3.2 (1.95–5.23)	-1.73×10^{-9} (-9.13×10^{-9}–0)
LumiraDx [b,c]	5.64 (1.33–10.9)	− 0.327 (− 0.943–− 0.0382)
LumiraDx [b,f]	12.1 (3.25–47.5)	− 1.05 (− 4.52–− 0.0284)
Omnia [b,c]	2.14 (1.35–3.19)	-1.81×10^{-9} (-2.88×10^{-8}–-4.02×10^{-10})
SCoV− 2 Ag Detect [b,c]	2.03 (1.34–3.42)	− 0.0537 (− 0.289–0)
Simoa [b,f]	4.17 (3.06–7.28)	− 0.0778 (− 0.352–0)
Sofia [b,c]	3.37 (1.61–6.71)	-8.89×10^{-10} (-1.12×10^{-8}–-1.53×10^{-10})
Sofia [c,d]	1.35 (0.646–2.22)	-9.23×10^{-10} (-1.12×10^{-8}–-2.27×10^{-10})
Sofia [c,e]	1.9 (1.27–2.71)	-8.98×10^{-10} (-4.68×10^{-9}–0)
Sofia 2 Flu + SARS [b,c]	4.77 (2.19–11.4)	− 0.6 (− 2.1–0)
Status COVID− 19/Flu [b,f]	2.93 (2.02–4.99)	− 0.135 (− 0.667–0)
Vitros [b,f]	1.39 (0.453–2.36)	-8×10^{-10} (-4.23×10^{-9}–-2.33×10^{-12})

[a] Peer-reviewed EUA data
[b] Data from EUA submission
[c] Anterior nasal swab
[d] Data from community testing
[e] Data from EUA submission and community testing
[f] Nasopharyngeal swab
[g] Mid-turbinate swab
