# Supplementary material for: Comparative analyses of eighteen rapid antigen tests and RT-PCR for COVID-19 quarantine and surveillance-based isolation
Source: Commun Med (Lond). 2022 Jul 9;2:84. doi: 10.1038/s43856-022-00147-y (PMC9271059; doi:10.1038/s43856-022-00147-y)
Supplement: Supplementary file 5 — Supplementary Data 3 [file 43856_2022_147_MOESM5_ESM.pdf]

Test	Fraction of RA tests in agreement with negative RT-PCR	Specificity (95% CrI)	Data Source
RT-PCR	N/A	99.90% (99.84%–99.95%) (12392/12404)	Community testing
BD Veritor [a]	212/213	99.43% (97.58%–99.91%)	EUA submission [b]
BinaxNOW [a]	338/343	98.45% (96.60%–99.43%)	EUA submission
BinaxNOW [a]	2004/2016	99.31% (98.91%–99.59%)	Community testing
BinaxNOW [a]	2342/2359	99.18% (98.75%–99.49%)	EUA submission and community testing
CareStart [a]	53/53	99.90% (94.40%–99.92%)	EUA submission
CareStart [d]	147/148	99.23% (96.50%–99.90%)	EUA submission
CareStart [a]	1243/1264	98.24% (97.38%–98.90%)	Community testing
CareStart [a]	1296/1317	98.31% (97.52%–98.89%)	EUA submission and community testing
Celltrion [d]	102/103	98.93% (95.06%–99.86%)	EUA submission
Clip COVID [a]	134/134	99.90% (97.52%–99.96%)	EUA submission
Ellume [e]	156/161	96.80% (93.11%–98.74%)	EUA submission
Liaison [a]	108/108	99.90% (96.84%–99.96%)	EUA submission
Liaison [d]	133/134	99.16% (96.16%–99.87%)	EUA submission
LumiraDx [a]	168/174	96.46% (92.83%–98.57%)	EUA submission
LumiraDx [d]	210/215	97.58% (94.73%–99.08%)	EUA submission
Omnia [a]	32/32	99.90% (90.58%–99.92%)	EUA submission
SCoV-2 Ag Detect [a]	257/257	99.90% (98.72%–99.95%)	EUA submission
Simoa [d]	38/38	99.90% (92.14%–99.92%)	EUA submission
Sofia [a]	179/179	99.90% (98.09%–99.96%)	EUA submission
Sofia [a]	1025/1041	98.37% (97.46%–99.03%)	Community testing
Sofia [a]	1204/1220	98.59% (97.77%–99.16%)	EUA submission and community testing
Sofia 2 Flu + SARS [a]	122/122	99.90% (97.55%–99.96%)	EUA submission
Status COVID-19/Flu [d]	76/76	99.90% (95.70%–99.96%)	EUA submission
VITROS [d]	75/75	99.90% (95.45%–99.97%)	EUA submission

[a] Anterior nasal swab
[b] Peer-reviewed EUA data
[c] Calculated based on both symptomatic and asymptomatic individuals
[d] Nasopharyngeal swab
[e] Mid-turbinate swab
