# Supplementary material for: Comparative analyses of eighteen rapid antigen tests and RT-PCR for COVID-19 quarantine and surveillance-based isolation
Source: Commun Med (Lond). 2022 Jul 9;2:84. doi: 10.1038/s43856-022-00147-y (PMC9271059; doi:10.1038/s43856-022-00147-y)
Supplement: Supplementary file 6 — Supplementary Data 4 [file 43856_2022_147_MOESM6_ESM.pdf]

Rapid antigen test	Quarantine required		Serial testing required	
	Exit test [a]	Entry and exit test [a]	Frequency [b]	Prob. of a false positive [c]
BD Veritor [d,e]	8 (8–8)	7 (7–8)	2 (2–2)	0.0389 (0.00845–0.152)
BinaxNOW [e,f]	8 (8–8)	7 (7–7)	2 (2–2)	0.104 (0.0428–0.21)
BinaxNOW [g,f]	8 (8–8)	7 (7–7)	2 (2–3)	0.0474 (0.0264–0.0751)
BinaxNOW [h,f]	8 (8–8)	7 (7–7)	2 (2–2)	0.0558 (0.0364–0.0833)
CareStart [e,f]	8 (8–8)	7 (7–7)	2 (2–2)	0.00675 (0.00407–0.33)
CareStart [e,i]	8 (8–8)	6 (6–7)	3 (2–3)	0.0355 (0.00716–0.188)
CareStart [f,g]	8 (8–8)	7 (7–7)	2 (2–2)	0.117 (0.0771–0.17)
CareStart [f,h]	8 (8–8)	7 (7–7)	2 (2–2)	0.112 (0.0739–0.164)
Celltrion DiaTrust [e,i]	8 (8–8)	6 (6–7)	3 (2–3)	0.0488 (0.00846–0.26)
Clip COVID [e,f]	8 (8–8)	7 (6–7)	3 (2–3)	0.00451 (0.00315–0.144)
Ellume [e,j]	8 (8–8)	6 (6–7)	3 (2–3)	0.141 (0.0628–0.357)
Liaison [e,f]	8 (8–8)	7 (6–7)	3 (2–3)	0.00451 (0.00325–0.175)
Liaison [e,i]	8 (8–8)	7 (6–7)	2 (2–3)	0.0575 (0.00863–0.221)
LumiraDx [e,f]	8 (8–8)	6 (6–7)	3 (2–3)	0.155 (0.0767–0.374)
LumiraDX [e,i]	8 (8–8)	6 (6–7)	3 (2–3)	0.108 (0.0473–0.28)
Omnia [e,f]	8 (8–8)	7 (7–7)	2 (2–2)	0.00675 (0.00419–0.478)
SCoV-2 Ag Detect [e,f]	8 (8–8)	7 (7–7)	2 (2–2)	0.00675 (0.00387–0.0857)
Simoa [e,i]	8 (8–8)	6 (6–7)	3 (2–3)	0.00451 (0.00323–0.393)
Sofia [e.f]	8 (8–8)	7 (6–7)	3 (2–3)	0.00451 (0.00329–0.113)
Sofia [g,f]	8 (8–8)	7 (7–8)	2 (1–2)	0.109 (0.0637–0.241)
Sofia [h,f]	8 (8–8)	7 (7–7)	2 (2–2)	0.0944 (0.0586–0.145)
Sofia 2 Flu+SARS [e,f]	8 (8–8)	7 (6–7)	3 (2–3)	0.00451 (0.00308–0.15)
Status COVID-19/Flu [e,i]	8 (8–8)	7 (6–7)	2 (2–3)	0.00675 (0.00363–0.243)
Vitros [e,i]	8 (8–8)	7 (7–8)	2 (1–2)	0.00675 (0.00402–0.284)

[a] Quarantine durations that are equivalent or better than a 7-day quarantine with an RT-PCR test conducted 24 h before exit.
[b] The minimum required testing frequency for serial testing such that the effective reproductive number is less than one.
[c] The probability of at least one false positive in a two-week period of serial testing under the minimum required testing frequency.
[d] Peer-reviewed
[e] Data from EUA submission
[f] Anterior nasal swab
[g] Data from community testing
[h] Combined data from EUA submission and community testing
[i] Nasopharyngeal swab
[j] Mid-turbinate swab
