# Supplementary material for: Comparative analyses of eighteen rapid antigen tests and RT-PCR for COVID-19 quarantine and surveillance-based isolation
Source: Commun Med (Lond). 2022 Jul 9;2:84. doi: 10.1038/s43856-022-00147-y (PMC9271059; doi:10.1038/s43856-022-00147-y)
Supplement: Supplementary file 7 — Supplementary Data 5 [file 43856_2022_147_MOESM7_ESM.pdf]

Rapid antigen test	Quarantine required		Serial testing required	
	Exit test [a]	Entry and exit test [a]	Frequency [b]	Prob. of a false positive [c]
BD Veritor [d,e]	9<br>(8–9)	9<br>(8–9)	2<br>(2–2)	0.0389<br>(0.00847–0.155)
BinaxNOW [e,f]	9<br>(8–9)	9<br>(8–9)	2<br>(2–2)	0.104<br>(0.0415–0.217)
BinaxNOW [g,f]	9<br>(8–9)	8<br>(8–9)	2<br>(2–3)	0.0474<br>(0.0255–0.0751)
BinaxNOW [h,f]	9<br>(8–9)	9<br>(8–9)	2<br>(2–2)	0.0558<br>(0.0357–0.0841)
CareStart [e,f]	9<br>(8–9)	9<br>(8–9)	2<br>(2–2)	0.00675<br>(0.00408–0.333)
CareStart [e,i]	8<br>(8–9)	8<br>(8–9)	3<br>(2–3)	0.0355<br>(0.00729–0.192)
CareStart [f,g]	9<br>(8–9)	9<br>(8–9)	2<br>(2–2)	0.117<br>(0.0752–0.173)
CareStart [f,h]	9<br>(8–9)	9<br>(8–9)	2<br>(2–2)	0.112<br>(0.0729–0.166)
Celltrion DiaTrust [e,i]	8<br>(8–9)	8<br>(8–9)	2<br>(2–3)	0.0723<br>(0.009–0.267)
Clip COVID [e,f]	8<br>(8–9)	8<br>(8–9)	2<br>(2–3)	0.00675<br>(0.00331–0.145)
Ellume [e,j]	8<br>(8–9)	8<br>(8–9)	3<br>(2–3)	0.141<br>(0.0642–0.363)
Liaison [e,f]	8<br>(8–9)	8<br>(8–9)	2<br>(2–3)	0.00675<br>(0.00314–0.174)
Liaison [e,i]	8<br>(8–9)	8<br>(8–9)	2<br>(2–3)	0.0575<br>(0.00868–0.222)
LumiraDx [e,f]	8<br>(8–9)	8<br>(8–9)	3<br>(2–3)	0.155<br>(0.0796–0.381)
LumiraDX [e,i]	8<br>(8–9)	8<br>(8–9)	3<br>(2–3)	0.108<br>(0.0489–0.287)
Omnia [e,f]	9<br>(8–9)	9<br>(8–9)	2<br>(2–2)	0.00675<br>(0.00444–0.481)
SCoV-2 Ag Detect [e,f]	9<br>(8–9)	9<br>(8–9)	2<br>(2–2)	0.00675<br>(0.00389–0.0871)
Simoa [e,i]	8<br>(8–9)	8<br>(8–9)	2<br>(2–3)	0.00675<br>(0.00337–0.398)
Sofia [e.f]	8<br>(8–9)	8<br>(8–9)	2<br>(2–3)	0.00675<br>(0.00323–0.113)
Sofia [g,f]	9<br>(8–9)	9<br>(8–9)	2<br>(1–2)	0.109<br>(0.0658–0.261)
Sofia [h,f]	9<br>(8–9)	9<br>(8–9)	2<br>(2–2)	0.0944<br>(0.0576–0.147)
Sofia 2 Flu+SARS [e,f]	8<br>(8–9)	8<br>(8–9)	2<br>(2–3)	0.00675<br>(0.00314–0.155)
Status COVID-19/Flu [e,i]	9<br>(8–9)	9<br>(8–9)	2<br>(2–3)	0.00675<br>(0.00362–0.243)
Vitros [e,i]	9<br>(8–9)	9<br>(8–9)	2<br>(1–2)	0.00675<br>(0.00406–0.308)

[a] Quarantine durations that are equivalent or better than a 7-day quarantine with an RT-PCR test conducted 24 h before exit.
[b] The minimum required testing frequency for serial testing such that the effective reproductive number is less than one.
[c] The probability of at least one false positive in a two-week period of serial testing under the minimum required testing frequency.
[d] Peer-reviewed
[e] Data from EUA submission
[f] Anterior nasal swab
[g] Data from community testing
[h] Combined data from EUA submission and community testing
[i] Nasopharyngeal swab
[j] Mid-turbinate swab
