# Supplementary material for: Comparative analyses of eighteen rapid antigen tests and RT-PCR for COVID-19 quarantine and surveillance-based isolation
Source: Commun Med (Lond). 2022 Jul 9;2:84. doi: 10.1038/s43856-022-00147-y (PMC9271059; doi:10.1038/s43856-022-00147-y)
Supplement: Supplementary file 8 — Supplementary Data 6 [file 43856_2022_147_MOESM8_ESM.pdf]

Rapid antigen test	Threshold based on infectivity at 5.6 days after symptom onset			Threshold based on infectivity at 10 days after symptom onset		
	Exit test [a]	Entry and exit test [a]	Frequency [b]	Exit test [a]	Entry and exit test [a]	Frequency [b]
BD Veritor [d,e]	0	0	0	0	0	0
BinaxNOW [e,f]	0	0	0	0	0	0
BinaxNOW [g,f]	0	0	0	0	0	0
BinaxNOW [h,f]	0	0	0	0	0	0
CareStart [e,f]	0	0	0	0	0	0
CareStart [e,i]	0	1	0	0	0	0
CareStart [f,g]	0	0	0	0	0	0
CareStart [f,h]	0	0	0	0	0	0
Celltrion DiaTrust [e,i]	0	1	0	0	0	0
Clip COVID [e,f]	0	0	− 1	0	0	0
Ellume [e,j]	0	0	0	0	0	0
Liaison [e,f]	0	0	− 1	0	0	0
Liaison [e,i]	0	0	0	0	0	0
LumiraDx [e,f]	0	1	0	0	0	0
LumiraDx [e,i]	0	0	0	0	0	0
Omnia [e,f]	0	0	0	0	0	0
SCoV-2 Ag Detect [e,f]	0	0	0	0	0	0
Simoa [e,i]	0	1	− 1	0	0	0
Sofia [e.f]	0	0	− 1	0	0	0
Sofia [h,f]	0	0	0	0	0	0
Sofia [g,f]	0	0	0	0	0	0
Sofia 2 Flu+SARS [e,f]	0	0	− 1	0	0	0
Status COVID-19/Flu [e,i]	0	0	0	0	0	0
Vitros [e,i]	0	0	0	0	0	0

[a] Quarantine durations that are equivalent or better than a 7-day quarantine with an RT-PCR test conducted 24 h before exit.
[b] The minimum required testing frequency for serial testing such that the effective reproductive number is less than one.
[c] The probability of at least one false positive in a two-week period of serial testing under the minimum required testing frequency.
[d] Peer-reviewed
[e] Data from EUA submission
[f] Anterior nasal swab
[g] Data from community testing
[h] Combined data from EUA submission and community testing
[i] Nasopharyngeal swab
[j] Mid-turbinate swab
