# Supplementary material for: Comparative analyses of eighteen rapid antigen tests and RT-PCR for COVID-19 quarantine and surveillance-based isolation
Source: Commun Med (Lond). 2022 Jul 9;2:84. doi: 10.1038/s43856-022-00147-y (PMC9271059; doi:10.1038/s43856-022-00147-y)
Supplement: Supplementary file 9 — Supplementary Data 7 [file 43856_2022_147_MOESM9_ESM.pdf]

Rapid antigen test	Quarantine required		Serial testing required	
	Exit test [a]	Entry and exit test [a]	Frequency [b]	Prob. of a false positive [c]
BD Veritor [d,e]	8 (8–8)	7 (7–8)	2 (1–2)	0.0389 (0.00815–0.159)
BinaxNOW [e,f]	8 (8–8)	7 (7–7)	2 (1–2)	0.104 (0.0387–0.244)
BinaxNOW [g,f]	8 (8–8)	7 (7–7)	2 (2–3)	0.0474 (0.0245–0.0761)
BinaxNOW [h,f]	8 (8–8)	7 (7–7)	2 (2–3)	0.0558 (0.0319–0.0882)
CareStart [e,f]	8 (8–8)	7 (7–7)	2 (1–2)	0.00675 (0.00403–0.345)
CareStart [e,i]	8 (8–8)	7 (6–7)	2 (2–3)	0.0528 (0.00736–0.196)
CareStart [f,g]	8 (8–8)	7 (7–7)	2 (1–2)	0.117 (0.0718–0.236)
CareStart [f,h]	8 (8–8)	7 (7–7)	2 (1–2)	0.112 (0.0686–0.223)
Celltrion DiaTrust [e,i]	8 (8–8)	7 (6–7)	2 (2–3)	0.0723 (0.00852–0.269)
Clip COVID [e,f]	8 (8–8)	7 (6–7)	2 (2–3)	0.00675 (0.00324–0.145)
Ellume [e,j]	8 (8–8)	7 (6–7)	2 (2–3)	0.204 (0.0669–0.371)
Liaison [e,f]	8 (8–8)	7 (6–7)	2 (2–3)	0.00675 (0.00316–0.174)
Liaison [e,i]	8 (8–8)	7 (6–7)	2 (2–3)	0.0575 (0.00851–0.222)
LumiraDx [e,f]	8 (8–8)	7 (6–7)	2 (2–3)	0.223 (0.0808–0.385)
LumiraDX [e,i]	8 (8–8)	6 (6–7)	2 (2–3)	0.158 (0.0499–0.291)
Omnia [e,f]	8 (8–8)	7 (7–7)	2 (1–2)	0.00675 (0.00439–0.497)
SCoV-2 Ag Detect [e,f]	8 (8–8)	7 (7–7)	2 (1–2)	0.00675 (0.0038–0.0925)
Simoa [e,i]	8 (8–8)	7 (6–7)	2 (2–3)	0.00675 (0.00336–0.401)
Sofia [e.f]	8 (8–8)	7 (6–7)	2 (2–3)	0.00675 (0.00325–0.113)
Sofia [g,f]	8 (8–8)	7 (7–8)	2 (1–2)	0.109 (0.0678–0.273)
Sofia [h,f]	8 (8–8)	7 (7–7)	2 (1–2)	0.0944 (0.0534–0.19)
Sofia 2 Flu+SARS [e,f]	8 (8–8)	7 (6–7)	2 (2–3)	0.00675 (0.00309–0.155)
Status COVID-19/Flu [e,i]	8 (8–8)	7 (6–7)	2 (2–3)	0.00675 (0.0035–0.242)
Vitros [e,i]	8 (8–8)	7 (7–8)	2 (1–2)	0.00675 (0.00409–0.337)

[a] Quarantine durations that are equivalent or better than a 7-day quarantine with an RT-PCR test conducted 24 h before exit.
[b] The minimum required testing frequency for serial testing such that the effective reproductive number is less than one.
[c] The probability of at least one false positive in a two-week period of serial testing under the minimum required testing frequency.
[d] Peer-reviewed
[e] Data from EUA submission
[f] Anterior nasal swab
[g] Data from community testing
[h] Combined data from EUA submission and community testing
[i] Nasopharyngeal swab
[j] Mid-turbinate swab
