# Supplementary material for: Comparative analyses of eighteen rapid antigen tests and RT-PCR for COVID-19 quarantine and surveillance-based isolation
Source: Commun Med (Lond). 2022 Jul 9;2:84. doi: 10.1038/s43856-022-00147-y (PMC9271059; doi:10.1038/s43856-022-00147-y)
Supplement: Supplementary file 11 — Reporting Summary [file 43856_2022_147_MOESM11_ESM.pdf]

nature portfolio

Corresponding author(s): Dr. Jeffrey Townsend

Last updated by author(s): Jun 17, 2022

Reporting Summary

Nature Portfolio wishes to improve the reproducibility of the work that we publish. This form provides structure for consistency and transparency in reporting. For further information on Nature Portfolio policies, see our Editorial Policies and the Editorial Policy Checklist.

Statistics

For all statistical analyses, confirm that the following items are present in the figure legend, table legend, main text, or Methods section.

n/a	Confirmed	
☐	☒	The exact sample size (*n*) for each experimental group/condition, given as a discrete number and unit of measurement
☒	☐	A statement on whether measurements were taken from distinct samples or whether the same sample was measured repeatedly
☐	☒	The statistical test(s) used AND whether they are one- or two-sided<br>*Only common tests should be described solely by name; describe more complex techniques in the Methods section.*
☒	☐	A description of all covariates tested
☒	☐	A description of any assumptions or corrections, such as tests of normality and adjustment for multiple comparisons
☐	☒	A full description of the statistical parameters including central tendency (e.g. means) or other basic estimates (e.g. regression coefficient) AND variation (e.g. standard deviation) or associated estimates of uncertainty (e.g. confidence intervals)
☐	☒	For null hypothesis testing, the test statistic (e.g. *F*, *t*, *r*) with confidence intervals, effect sizes, degrees of freedom and *P* value noted<br>*Give P values as exact values whenever suitable.*
☒	☐	For Bayesian analysis, information on the choice of priors and Markov chain Monte Carlo settings
☒	☐	For hierarchical and complex designs, identification of the appropriate level for tests and full reporting of outcomes
☒	☐	Estimates of effect sizes (e.g. Cohen's *d*, Pearson's *r*), indicating how they were calculated

Our web collection on statistics for biologists contains articles on many of the points above.

Software and code

Policy information about availability of computer code

Data collection	Web plot Digitizer (Version 4.5) was used to extract data points for the baseline infectivity profile and the distribution for the incubation period.
Data analysis	MATLAB R2019b was used to conduct the simulations and analyze the data. The corresponding code is deposited in an on-line repository: Wells, C.R. et al. WellsRC/Comparative-analyses-FDA-EUA-rapid-antigen-tests-and-RT-PCR-for-COVID-19: MATLAB Code: Comparative analyses of eighteen rapid antigen tests and RT-PCR for COVID-19 quarantine and surveillance-based isolation (v1.0.0). Zenodo. https://doi.org/10.5281/zenodo.6518442 (2022).

For manuscripts utilizing custom algorithms or software that are central to the research but not yet described in published literature, software must be made available to editors and reviewers. We strongly encourage code deposition in a community repository (e.g. GitHub). See the Nature Portfolio guidelines for submitting code & software for further information.

Data

Policy information about availability of data

All manuscripts must include a data availability statement. This statement should provide the following information, where applicable:

- Accession codes, unique identifiers, or web links for publicly available datasets
- A description of any restrictions on data availability
- For clinical datasets or third party data, please ensure that the statement adheres to our policy

All data generated, analysed, and used to build the graphs and tables in this study are available in the online Zenodo repository (WellsRC/Comparative-analyses-FDA-EUA-rapid-antigen-tests-and-RT-PCR-for-COVID-19: MATLAB Code: Comparative analyses of eighteen rapid antigen tests and RT-PCR for COVID-19 quarantine and surveillance-based isolation (v1.0.0). Zenodo. https://doi.org/10.5281/zenodo.6518442 (2022)) or presented in the published article, supplementary

information, and supplementary datasets. The RT-PCR testing dataset used to infer the temporal RT-PCR diagnostic sensitivity is provided from the published article by Hellewell et al. (Estimating the effectiveness of routine asymptomatic PCR testing at different frequencies for the detection of SARS-CoV-2 infections. BMC Med 19, 106 (2021). https://doi.org/10.1186/s12916-021-01982-x). The PPA datasets used in the inference of the temporal diagnostic sensitivity of each rapid antigen test are found in Supplementary Data 1. The specificity of each test is summarized in Supplementary Data 3. The cycle times for rapid antigen test false negatives and true positives for the paired testing of BD Veritor and RT-PCR are located in Supplementary Table 3. Source data for all graphs and other tables are available from the Zenodo repository (WellsRC/Comparative-analyses-FDA-EUA-rapid-antigen-tests-and-RT-PCR-for-COVID-19: MATLAB Code: Comparative analyses of eighteen rapid antigen tests and RT-PCR for COVID-19 quarantine and surveillance-based isolation (v1.0.0). Zenodo. https://doi.org/10.5281/zenodo.6518442 (2022)). All other data are available from the corresponding author [JPT] on reasonable request.

Field-specific reporting

Please select the one below that is the best fit for your research. If you are not sure, read the appropriate sections before making your selection.

☒ Life sciences ☐ Behavioural & social sciences ☐ Ecological, evolutionary & environmental sciences

For a reference copy of the document with all sections, see nature.com/documents/nr-reporting-summary-flat.pdf

Life sciences study design

All studies must disclose on these points even when the disclosure is negative.

Sample size	No sample size calculation was conducted. The sample sizes were based on the available data of groups of crew members being tested 22 November 2020 to 17 January 2021, 2 March 2021 to 22 May 2021, and 5 March 2021 to 24 May 2021.
Data exclusions	There were no data exclusions.
Replication	The data obtained was dependent on the stage of the epidemic and the background prevalence of COVID-19 in the population. Thus, the study would be difficulty to reproduce; a similar study could be conducted at a subsequent date.
Randomization	Randomization was not relevant to our study. A RT-PCR and rapid antigen test were conducted on the same days over the course of quarantine. The serial testing study, testing was conducted with the BD Veritor test on days 3-6-9 for one group and 2-5-8 for another. These testing sequences were informed by the modeling analysis.
Blinding	Blinding was not relevant to our study as all individuals were required to undergo quarantine, and undergo serial testing after the test-and-fly

Reporting for specific materials, systems and methods

We require information from authors about some types of materials, experimental systems and methods used in many studies. Here, indicate whether each material, system or method listed is relevant to your study. If you are not sure if a list item applies to your research, read the appropriate section before selecting a response.

Materials & experimental systems

n/a	Involved in the study	
☒	☐	Antibodies
☒	☐	Eukaryotic cell lines
☒	☐	Palaeontology and archaeology
☒	☐	Animals and other organisms
☐	☒	Human research participants
☒	☐	Clinical data
☒	☐	Dual use research of concern

Methods

n/a	Involved in the study	
☒	☐	ChIP-seq
☒	☐	Flow cytometry
☒	☐	MRI-based neuroimaging

Human research participants

Policy information about studies involving human research participants

Population characteristics	November 2020 to 17 January 2021:<br>818 tests were conducted on entry to quarantine, 726 on day three and 675 tested on day four of quarantine with both RT-PCR and BD Veritor. These numbers will vary by Day as if a positive PCR the person is removed from further testing and out of quarantine pool; if an individual develops any symptoms, they are out of the quarantine pool; if work demands offshore change the staffing requirements can change; people can leave for personal reasons etc.—hence it is not the same number of paired tests across all days Corresponded with an observed rise in community transmission across Texas and Louisiana, the overwhelming residence for the majority of the offshore platform workers.<br>2 March 2021 to 22 May 2021:<br>1373 tests were conducted across a maximum of 458 individuals in the offshore oil rig setting. 457 underwent testing on all three days.<br>5 March 2021 to 24 May 2021

341 tests were conducted across a maximum of 124 individuals in the offshore oil rig setting. 96 individuals underwent testing on all three days.

Recruitment	Recruitment was based on crew members entering quarantine after entering a pre-screening process that filtered symptomatic individuals and those with recent exposure.
Ethics oversight	During quarantine, a positive RT-PCR led to removal of the individual from the quarantine environment, isolation for 10 days with medical follow-up. Afterward, workers could return to work after two negative RT-PCR tests.<br><br>All testing was conducted by the platform medic using the BD Veritor kit and reader. Any positive individual was isolated pending helicopter transfer (typically within 12–24 hours) to the established onshore medical facility, whereupon an RT-PCR nasal swab was obtained and sent to a commercial laboratory. A positive antigen test was considered to be a false positive if the follow-up RT-PCR was negative.<br><br>Conducting this study of the onshore and offshore testing of the oil platform employees, and the use of the resulting data, was approved by the Human Participants Review Sub-Committee, York University's Ethics Review Board (Certificate Number: 2021-003). All employees that participated in the study provided informed consent.<br><br>Ethical approval was not required for the datasets for RT-PCR sensitivity, PPA for each test and specificity of each test because they are available in the public domain.

Note that full information on the approval of the study protocol must also be provided in the manuscript.
